# Exploring the Potential Mechanism of Artemisinin and Its Derivatives in the Treatment of Osteoporosis Based on Network Pharmacology and Molecular Docking

**DOI:** 10.1155/2022/3976062

**Published:** 2022-12-22

**Authors:** Yujie Ma, Haixia Liu, Xinyue Lu, Changheng Song, Yin Cheng, Yuhan Wang, Pei Li, Yanjing Chen, Zhiguo Zhang

**Affiliations:** Institute of Basic Theory, China Academy of Chinese Medical Sciences, Beijing 100700, China

## Abstract

**Objective:**

This study is aimed at predicting and contrasting the mechanisms of artemisinin (ARS), dihydroartemisinin (DHA), artesunate (ART), artemether (ARM), and arteether (ARE) in the treatment of osteoporosis (OP) using network pharmacology and molecular docking.

**Methods:**

The targets of ARS, DHA, ART, ARM, and ARE were obtained from the SwissTargetPrediction. The targets related to OP were obtained from the TTD, DrugBank, Genecards, and DisGeNet databases. Then, the anti-OP targets of ARS, DHA, ART, ARM, and ARE were obtained and compared using the Venn diagram. Afterward, the protein-protein interaction (PPI) networks were built using the STRING database, and Cytoscape was used to select hub targets. Moreover, molecular docking validated the binding association between five molecules and hub targets. Finally, GO enrichment and KEGG pathway enrichment were conducted using the DAVID database. The common pathways of five molecules were analysed.

**Results:**

A total of 28, 37, 36, 27, and 33 anti-OP targets of ARS, DHA, ART, ARM, and ARE were acquired. EGFR, EGFR, CASP3, MAPK8, and CASP3 act as the top 1 anti-OP targets of ARS, DHA, ART, ARM, and ARE, respectively. MAPK14 is the common target of five molecules. All five molecules can bind well with these hubs and common targets. Meanwhile, functional annotation showed that MAPK, Serotonergic synapse, AMPK, prolactin, and prolactin signaling pathways are the top 1 anti-OP pathway of ARS, DHA, ART, ARM, and ARE, respectively. IL-17 signaling pathway and prolactin signaling pathway are common anti-OP pathways of five molecules. Besides, GO enrichment showed five biological processes and three molecular functions are common anti-OP mechanisms of five molecules.

**Conclusion:**

ARS, DHA, ART, ARM and ARE can treat OP through multi-targets and multi pathways, respectively. All five molecules can treat OP by targeting MAPK14 and acting on the IL-17 and prolactin signaling pathways.

## 1. Introduction

Osteoporosis (OP) is a bone metabolic disease common in the elderly [1]. OP patients have a poor quality of life due to chronic pain and deformed spines [2]. The rising number of fractures caused by OP leads to substantial morbidities, mortality, and expensive healthcare costs [3]. Despite immense treatment advances, concerns regarding long-term efficacy and numerous side effects make it urgent to find new, effective anti-OP drugs [4].

Artemisinin (ARS) is the first-line antimalarial drug acquired from *Artemisia annua L*, which has several derivatives such as dihydroartemisinin (DHA), artesunate (ART), artemether (ARM), and arteether (ARE) [5, 6]. In ancient medical books, A*rtemisia annua L* is mentioned to improve OP symptoms, including limb pain and joint inflexibility. Therefore, the anti-OP effect of ARS and its derivatives has gained wide attention. *In vivo*, *Artemisia annua* ethanol extract, ARS, and DHA can inhibit bone loss in ovariectomized mice [7, 8]. DHA and ART also prevent lipopolysaccharide (LPS)-induced bone loss [9]. To further explore the mechanism, the effects of ARS and its derivatives on osteoblast and osteoclast were studied *in vitro*. ARS, DHA, ART, and ARM can impair RANKL-induced osteoclast differentiation by hampering the expression of NFATc1 [7, 8, 10–12]. Besides, DHA can suppress osteoclastogenesis by suppressing the NF-*κ*B activation and controlling the mitochondria-dependent apoptosis pathway [13]. ART can inhibit osteoclastogenesis via the miR-503/RANK axis and enhance osteoblast differentiation by miR-34a/DKK1 axis [11, 14]. Meanwhile, current studies only focused on the anti-OP mechanism of ARM in regulating the MAPK (ERK, JNK, p-38) pathway [12].

As mentioned above, ARS and its derivatives can play the anti-OP role through similar and unique mechanisms. A comprehensive and systematic mechanism for treating OP is still not widely reported. This study investigates the potential mechanism of ARS and its derivatives in treating OP using network pharmacology, which provides a reference for further experimental research.

## 2. Methods

### 2.1. Identification of the Basic Information of ARS and Its Derivatives

The canonical simplified molecular input line entry specification (SMILES) and the structure of ARS and its derivatives were obtained from PubChem database (https://pubchem.ncbi.nlm.nih.gov/). The SMILES were input into SwissTargetPrediction (http://www. swisstargetprediction.ch/) to obtain potential targets (probability >0) of ARS and its derivatives. Homo sapiens was selected as the target organism.

### 2.2. Screening OP-Associated Targets

OP-connected genes were acquired from these online databases: Therapeutic Target Database (TTD http://database.idrb.cqu.edu.cn/TTD/), the DrugBank database (https://www.drugbank.ca/), Genecards (Relevance score > mean, http://www.genecards.org), and DisGeNet (https://www.disgenet.org/). The UniProtKB ID was used to verify all targets.

### 2.3. Venn Diagram Analysis

The overlapping of drug-related and OP-associated targets might be the potential targets for drugs against OP. Anti-OP targets of ARS, DHA, ART, ASM, and ARE were acquired by Venn online tool (http://jvenn.toulouse.inra.fr/app/example.html). Moreover, a Venn diagram compared the potential anti-OP targets of five molecules.

### 2.4. PPI Network Construction and Hub Targets Analysis

Protein-protein interaction (PPI) networks were constructed to obtain the interaction between targets by inputting anti-OP targets of each molecule into the STRING database, respectively, (https://cn.string-db.org/). The protein type was set to Homo sapiens, and the minimum required interaction score was medium (0.4). The PPI results were moved to Cystoscope (3.7.2) to screen the hub targets with a high degree.

### 2.5. Molecular Docking

The five molecules' common and hub targets were verified by docking with the five molecules. The structure data files (SDFs) of five molecules were acquired from PubChem and converted to protein data bank (PDB) files via open Babel. The 3D structures of target proteins were acquired from the PDB (http://www.pdb.org/). Autodock tools software aids in the process of the receptors and ligands. Autodock Vina was used for molecular docking and to acquire binding energy. Finally, the graphical software PyMOL was used to illustrate the docking of the receptor and ligand.

### 2.6. Enrichment Analysis of GO and KEGG Pathways

Gene Ontology (GO) and Kyoto Encyclopedia of Genes and Genomes (KEGG) pathway enrichment of each molecule was conducted by the Database for Annotation, Visualization and Integrated Discovery (DAVID, version 6.8, https://david.ncifcrf.gov/). A *P* value less than 0.05 were considered significantly enriched. The bubble diagrams were constructed to analyze the top 10 biological pathways (BPs), cell localization (CC), molecular function (MF), and the top 20 KEGG pathways that were significantly enriched. The common terms of five molecules of the specific terms of each molecule were obtained in excel.

### 2.7. Common Pathways Analysis

The maps of the common KEGG pathways were acquired from the KEGG database (https://www.genome.jp/kegg/). Different colors were used to represent targets of different molecules. The genes enriched by five molecules in the common pathway were presented on one map.

## 3. Results

### 3.1. Basic Information on ARS and Its Derivatives

The canonical SMILES and molecule structure of ARS, DHA, ART, ARM, and ARE acquired from PubChem are shown in Table 1. The structure of ARS and its derivatives are particular for the internal peroxide bridge [5]. The SMILES formats of five molecules were input into SwissTargetPrediction to predict corresponding targets. As a result, 76 targets were predicted for ARS, 95 targets for DHA, 88 targets for ART, 89 targets for ARM, and 98 targets for ARE.

### 3.2. The Potential anti-OP Targets of ARS and Its Derivatives

**Figure 1:**
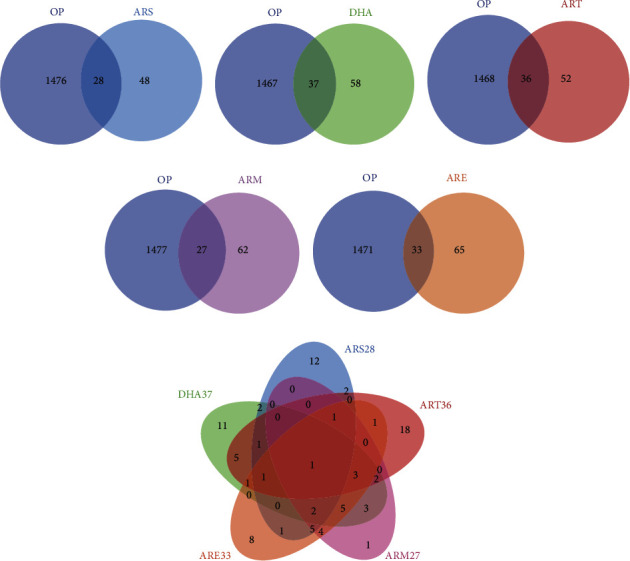
The Venn diagram of targets for treating OP. (a–e) The Venn diagram of targets of ARS-OP (a), DHA-OP (b), ART-OP (c), ARM-OP (d), ARE-OP (e). (f) The Venn diagram of anti-OP targets of five molecules.

1504 OP-related targets were obtained from TTD, Genecards, DrugBank, and DisGeNet databases using the keyword “osteoporosis”. The overlapping targets of molecules and OP were considered as targets for treating OP. 28, 37, 36, 27, and 33 targets of ARS, DHA, ART, ARM, and ARE were identified to treat OP. The Venn diagrams are generated as Figures 1(a)–1(e). The targets of five molecules against OP were compared. Five molecules share MAPK14. The details of common and specific anti-OP targets of five molecules are shown in Figure 1(f) and Table 2.

### 3.3. PPI Network Construction and Hub Targets Analysis

**Figure 2:**
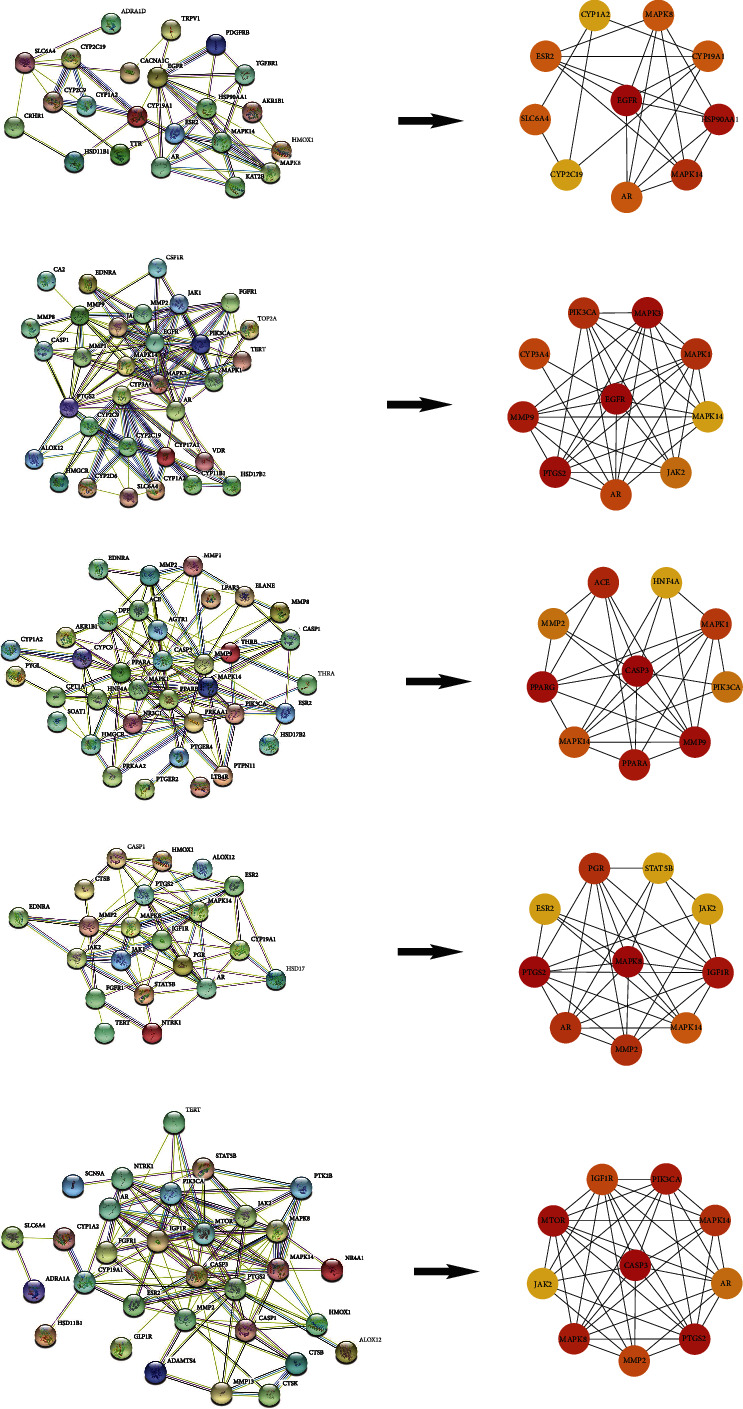
PPI networks and hub targets' networks. (a) ARS, (b) DHA, (c) ART, (d) ARM, and (e) ARE.

As shown in Figure 2, the PPI networks exhibit the interaction between targets. Cystoscope generates the new simple networks and screens the top 10 hub targets of each molecule. Based on the new network, EGFR, HSP90AA1, and MAPK14 are the top 3 anti-OP targets of ARS. EGFR, MAPK3, and PTGS2 are the top 3 anti-OP targets of DHA. CASP3, MMP9, and PPARG are the top 3 anti-OP targets of ART. MAPK8, PTGS2, and IGF1R are the top 3 anti-OP targets of ARM. CASP3, MTOR, and PTGS2 are the anti-OP effect of ARE.

### 3.4. Molecular Docking

**Figure 3:**
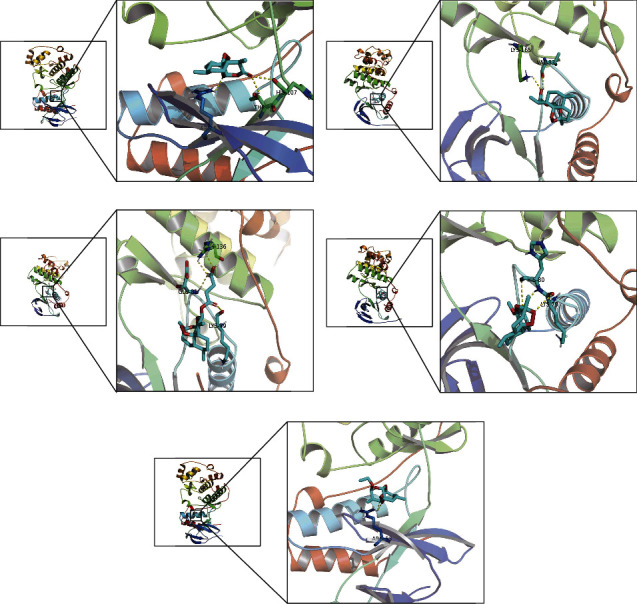
Schematic diagram on the docking of MAPK14 with ARS (a), DHA (b), ART (c), ARM (d), and ARE (e).

MAPK14 is the only common target of 5 molecules. EGFR, EGFR, CASP3, MAPK8, and CASP3 are the top1 hub anti-OP targets of ARS, DHA, ART, ARM, and ARE, respectively. Molecular docking is conducted to investigate the binding of five molecules with EGFR, CASP3, MAPK8, and MAPK14.  Table 3 shows the calculated binding energy.  Figure 3 shows the docking visualization of five molecules and MAPK14. The binding energy of less than -5 kcal/mol indicates a stable binding between the ligands and receptors [15, 16]. The results reveal that five molecules can bind well with MAPK14 and hub targets.

### 3.5. GO Enrichment

**Figure 4:**
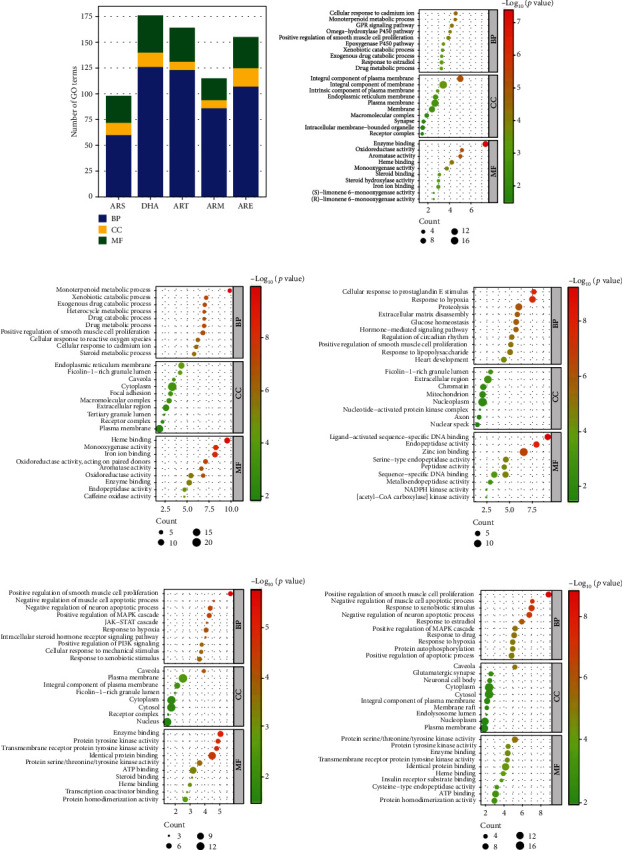
GO enrichment analysis. (A) The number of enriched GO terms. (b–f) Top GO enriched terms of ARS, DHA, ART, ARM, and ARE in treating OP.

GO enrichment of five molecules was performed, respectively, to determine the anti-OP mechanism of each molecule.  Figure 4(a) shows that ARS-OP targets were significantly enriched into 60 BP terms, 12 CC terms, and 26 MF terms; DHA-OP targets were enriched into 126 BP terms, 14 CC terms, and 36 MF terms; ART-OP targets were enriched into 123 BP terms, 8 CC terms, and 33 MF terms; ARM-OP targets were enriched into 86 BP terms, 8 CC terms, and 21 MF terms; ARE-OP targets were enriched into 107 BP terms, 18 CC terms, and 30 MF terms.  Figure 4(b)–4(f) displays the bubble chart of each category's top 10 GO terms.

The common GO terms can uncover the common mechanism of five molecules. A total of 5 BP and 3 MF were identified, and no CC was shared by all five molecules (Table 4).

### 3.6. KEGG Enrichment

**Figure 5:**
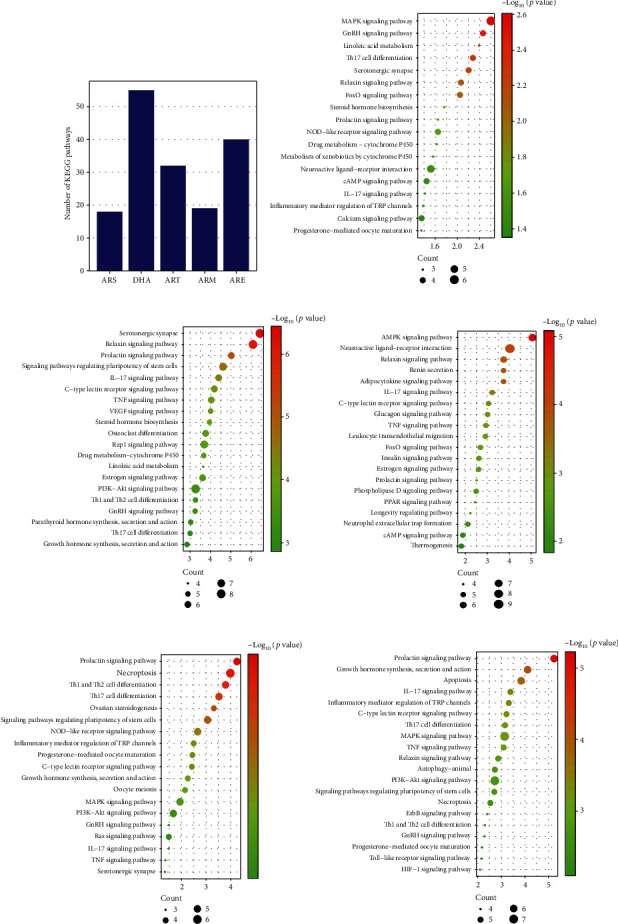
KEGG enrichment analysis. (a) The number of enriched KEGG pathways. (b–f) Top KEGG enriched pathways of ARS, DHA, ART, ARM, and ARE in treating OP.

KEGG pathways enrichment was conducted to predict the potential anti-OP pathways of five molecules, respectively. The KEGG pathway involved in human disease section was removed because OP was caused by basic biological dysfunctions [16].  Figure 5(a) shows 18 pathways were significantly enriched from ARS-OP targets, 55 pathways from DHA-OP targets, 32 pathways from ART-OP targets, and 19 pathways from ARM-OP targets, 40 pathways from ARE-OP targets. 10 pathways with minimum *P* values were plotted in the bubble chart, as shown in Figure 5(b)–5(f).

On the other hand, the prolactin signaling pathway and IL-17 signaling pathway were found to be shared by five molecules, a common anti-OP mechanism of five molecules. In addition, specific pathways of each molecule have been found. The calcium signaling pathway is the ARS-specific pathway, and apoptosis is the ARE-specific pathway. DHA has 15 specific pathways, such as parathyroid hormone synthesis, secretion and action, sphingolipid signaling, and arachidonic acid metabolism. ART has 8 specific pathways, such as renin secretion, glucagon signaling pathway, and thermogenesis. More details on the common and specific KEGG pathways are shown in Table 5.

### 3.7. Common Pathways Analysis

**Figure 6:**
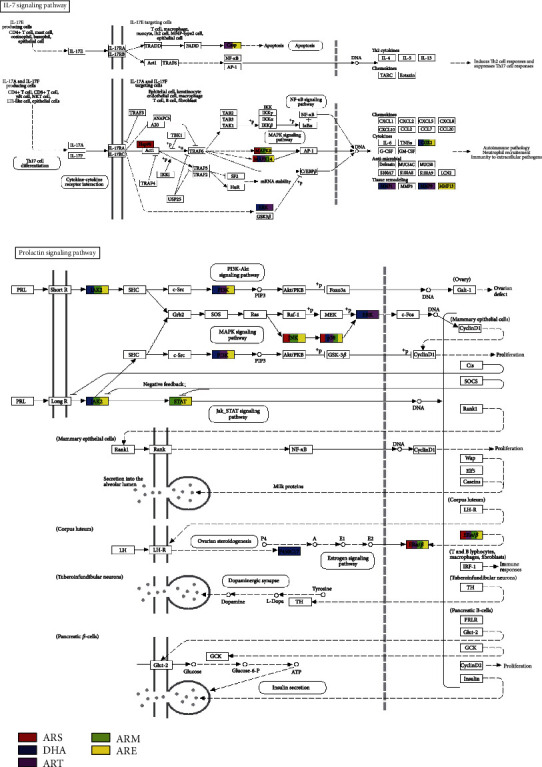
The targets of five molecules in the common pathways. (a) The targets of ARS, DHA, ART, ARM, and ARE in the IL-17 signaling pathways. (b) The targets of ARS, DHA, ART, ARM, and ARE in the prolactin signaling pathways.

All five molecules exhibit an anti-OP effect by prolactin signaling pathway and IL-17 signaling pathway, acting on different genes of the two pathways. In the IL-17 signaling pathway, ARS target at HSP90AA1, MAPK8, and MAPK14; DHA target at MMP1, MAPK1, MAPK14, PTGS2, MMP9, and MAPK3; ART target at MMP1, CASP3, MAPK1, MAPK14, and MMP9; ARM target at MAPK8, MAPK14, and PTGS2; and ARE target at MAPK8, MMP13, CASP3, MAPK14, and PTGS2. In the prolactin signaling pathway, ARS target at MAPK8, MAPK14, and ESR2; DHA target at PIK3CA, MAPK1, JAK2, MAPK14, CYP17A1, and MAPK3; ART target at PIK3CA, MAPK1, MAPK14, and ESR2; ARM target at STAT5B, MAPK8, JAK2, MAPK14, and ESR2; and ARE target at STAT5B, MAPK8, PIK3CA, JAK2, MAPK14, and ESR2.  Figure 6 illustrates the location of each target, where rectangles of different colors represent the target genes of different molecules.

## 4. Discussion

ARS and its derivatives have been used to treat many diseases, such as cancers, viral infections, inflammatory, and autoimmune diseases [17–19]. ARS and its derivatives can improve bone metabolism *in vivo* and *in vitro*, but detailed mechanisms are unclear [7–14]. Network pharmacology, a systems biology-based methodology, is used to identify the anti-OP mechanism of ARS and its derivatives entirely [20]. Moreover, the anti-OP mechanism of these molecules was compared.

It is found that ARS, DHA, ART, ARM, and ARE act in an anti-OP role through multi targets and multi pathways, respectively. In our study, EGFR is the most noticeable anti-OP target of ARS and DHA, CASP3 is the most important anti-OP target of ART and ARE, and MAPK8 is the most promising target of ARM. Previous studies have reported the importance of EGFR, CASP3, and MAPK8 in the pathogenesis of OP. EGFR can regulate the proliferation and differentiation of osteoblast and induce osteoclast differentiation by upregulating RANKL expression [21, 22]. The degeneration of cortical bone caused by aging is also controlled by EGFR signaling [23]. CASP3, the crucial enzyme in the execution phase of apoptosis, is abnormally expressed in the OP model. It is essential for self-renewal and osteogenic/adipogenic differentiation of MSCs [24–26]. DHA can increase the expression of CASP3 during LPS-induced osteoclastogenesis [13]. MAPK8 can regulate osteoblast autophagy and mitophagy, promoting extracellular matrix mineralization [27–29]. Therefore, EGFR, CASP3, and MAPK8 were focused on the next experiments exploring the anti-OP mechanism of ARS and its derivatives.

KEGG analysis suggested that the MAPK signaling pathway, serotonergic synapse, AMPK signaling pathway, prolactin signaling pathway, and prolactin signaling pathway are the top 1 anti-OP pathway of ARS, DHA, ART, ARM, and ARE, respectively. These pathways are known as OP-related pathways. MAPK signaling pathway is a classical signaling pathway for regulating bone metabolism [30]. Experimental studies have found that DHA and ARM can restore bone loss by the MAPK signaling pathway [12, 31]. Serotonergic synapses can secrete serotonin, a neurotransmitter that increases bone formation and decreases bone resorption [32, 33]. As an intracellular sensor for regulating the energy balance, AMPK is a potential therapeutic target for OP. AMPK can determine the differentiation of mesenchymal progenitor cells into adipocytes or osteoblasts by regulating the expression of Runx2 and PPARG, inhibiting the formation of osteoclasts and bone resorption through NFATc1 [34]. Clinical and animal experiments have proved that abnormal prolactin level is related to bone metabolism disorder. Further studies reveal that prolactin can indirectly affects bone remodeling by regulating sex hormone levels [35]. Further experimental validation is required to investigate the effect of ARS and its derivatives on these pathways.

There are a few common anti-OP mechanisms among the five molecules. MAPK14 is the common target of five molecules, regulating the expression of OPG and promoting the proliferation and differentiation of osteoclast progenitors [36, 37]. All five molecules can regulate the module function of enzyme binding, identical protein binding, and MAP kinase activity to reduce bone loss. These molecules can improve bone metabolism by five biological processes, including positive regulation of smooth muscle cell proliferation, positive regulation of gene expression, positive regulation, apoptotic process, signal transduction, and intracellular signal transduction. KEGG analysis shows that IL-17 and prolactin signaling pathways are common pathways of five molecules, and both are vital in the progression of OP. The IL-17 signaling pathway is a classical way that mediates bone and immune cells. Postmenopausal women's low bone mass density is associated with high plasma IL-17 level [38]. IL-17A, an important member of the IL-17 family, plays a dual role in osteoclasts and osteoblasts [39, 40]. The prolactin signaling pathway can regulate sex hormone levels, which are crucial in bone metabolism [35]. Five molecules can act on the two pathways together but at different targets. These common anti-OP mechanisms may be acquired from the same structures of ARS and its derivatives.

Interestingly, among the five molecules, DHA can act on the most targets, regulate the most GO MF, and involve the most GO BP and KEGG pathways. Besides, ARS, DHA, ART, ARM, and ARE have specific targets, BP terms, and KEGG pathways.

There were a few shortcomings in this study. Due to the limitations of network pharmacology, the dose-effect relationships of ARS, DHA, ART, ARM, and ARE are required to explore in additional studies. The anti-OP mechanisms of five molecules also need further experimental validation. However, this study not only provides a basis for subsequent experimental verification but provides an example for exploring the mechanism of similar compounds using network pharmacology.

## 5. Conclusion

In conclusion, ARS, DHA, ART, ARM, and ARE act in an anti-OP role through multitargets and multipathways, respectively. DHA is a prominent molecule due to its many targets and pathways. All five molecules can treat OP by targeting MAPK14 and acting on the IL-17 and prolactin signaling pathways.

## Figures and Tables

**Table 1:** Basic information on ARS and its derivatives.

PubChem CID	Molecular name	Canonical SMILES	Molecular structure
68827	ARS	CC1CCC2C(C(=O)OC3C24C1CCC(O3)(OO4)C)C	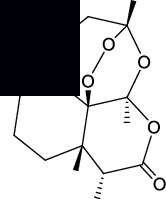
3000518	DHA	CC1CCC2C(C(OC3C24C1CCC(O3)(OO4)C)O)C	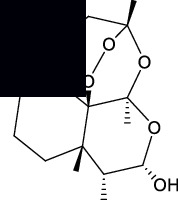
6917864	ART	CC1CCC2C(C(OC3C24C1CCC(O3)(OO4)C)OC(=O)CCC(=O)O)C	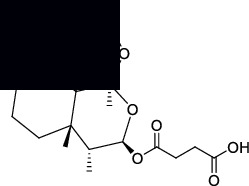
68911	ARM	CC1CCC2C(C(OC3C24C1CCC(O3)(OO4)C)OC)C	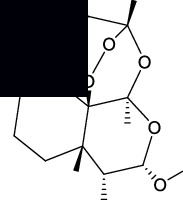
3000469	ARE	CCOC1C(C2CCC(C3C24C(O1)OC(CC3)(OO4)C)C)C	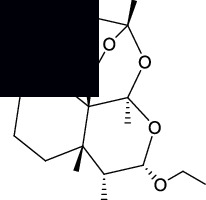

**Table 2:** The common targets and specific targets of five molecules against OP.

Molecules	Targets
Common	MAPK14
ARS specific	CASR, TTR, TGFBR1, ALPL, TRPV1, CACNA1C, PDGFRB, CCR3, CRHR1, ADRA1D, HSP90AA1, KAT2B
DHA specific	CYP3A4, MAPK3, CSF1R, VDR, CYP17A1, ASAH1, CYP11B1, CYP2D6, TOP2A, BRS3, LGMN
ART specific	NR3C1, LTB4R, DPP4, ACE, PTGER4, PPARG, PTGER2, THRB, ELANE, THRA, AGTR1, HNF4A, LPAR3, PPARA, PTPN11, PRKAA1, PRKAA2, SOAT1
ARM specific	PGR
ARE specific	PTK2B, MMP13, CTSK, SCN9A, ADAMTS4, ADRA1A, NR4A1, MTOR

**Table 3:** The docking energy of five molecules binding to common and hub targets.

Molecular name	Targets	PDB ID	Docking score (kcal/mol)
ARS	MAPK14	5ETI	-6.8
DHA	MAPK14	5ETI	-6.9
ART	MAPK14	5ETI	-7.6
ARM	MAPK14	5ETI	-6.3
ARE	MAPK14	5ETI	-6.2
ARS	EGFR	5GTY	-8.7
DHA	EGFR	5GTY	-8.7
ART	CASP3	2 J30	-6.3
ARM	MAPK8	3PZE	-6.0
ARE	CASP3	2 J30	-6.6

**Table 4:** The common GO terms of five molecules.

GO	Common GO terms
BP	Positive regulation of smooth muscle cell proliferation, positive regulation of gene expression, positive regulation of apoptotic process, signal transduction, intracellular signal transduction
CC	None
MF	Enzyme binding, identical protein binding, MAP kinase activity

**Table 5:** The common KEGG pathways and specific KEGG pathways of five molecules against OP.

Molecules	KEGG pathways
Common	IL-17 signaling pathway, prolactin signaling pathway
ARS specific	Calcium signaling pathway
DHA specific	Parathyroid hormone synthesis, secretion and action, sphingolipid signaling pathway, arachidonic acid metabolism, Adherens junction, T cell receptor signaling pathway, aldosterone-regulated sodium reabsorption, cholinergic synapse, regulation of actin cytoskeleton, platelet activation, metabolic pathways, retrograde endocannabinoid signaling, oxytocin signaling pathway, cellular senescence, retinol metabolism, B cell receptor signaling pathway
ART specific	Renin secretion, glucagon signaling pathway, thermogenesis, PPAR signaling pathway, thyroid hormone signaling pathway, natural killer cell mediated cytotoxicity, Apelin signaling pathway, mTOR signaling pathway
ARM specific	None
ARE specific	Apoptosis

## Data Availability

The data used to support this study are available from the corresponding author upon reasonable request.
